# Root Cap Cuticles Confer a Transient, Penetration‐Optimised Phase During Early Seedling Establishment

**DOI:** 10.1111/pce.70622

**Published:** 2026-05-22

**Authors:** Woo‐Taek Jeon, Jeong‐A. Kim, Ahyeon Cheon, Yesol Shin, Shawn S. Y. Lee, Yuree Lee

**Affiliations:** ^1^ School of Biological Sciences Seoul National University Seoul Republic of Korea; ^2^ Department of Plant Science, College of Agriculture and Life Science Seoul National University Seoul Republic of Korea; ^3^ Research Center for Plant Plasticity Seoul National University Seoul Republic of Korea; ^4^ Plant Genomics and Breeding Institute Seoul National University Seoul Republic of Korea

**Keywords:** Arabidopsis, gravitropism, mechanical stress, root cap cuticle, root cap detachment, root penetration

## Abstract

Roots emerging from seeds encounter substantial mechanical resistance during soil entry. A transient root cap cuticle (RCC) forms early in development, yet its contribution to root tip function during this mechanically challenging transition remains unclear. Here, we define an early functional window in which the root tip is transiently optimised for establishment under soil‐imposed constraints, and show that this window depends on the RCC. Consistent with a protective role, RCC‐deficient seedlings were less efficient at traversing dense substrates and accumulated greater mechanical damage at the tip. Notably, RCC function extended beyond protection: when roots were grown on agar surfaces, RCC deficiency accelerated detachment of the outermost root cap cell layer and disrupted its layer integrity, indicating an additional role in maintaining coordinated root cap turnover. This early window faded with seedling age, preceding RCC loss, and coincided with a developmental reduction in gravitropic responsiveness, thereby defining a restricted period for directional control. RCC‐defective mutants exhibited impaired gravitropism across stages, whereas hydrotropic responses were largely unaffected, linking RCC function specifically to gravitropic regulation. Together, these results delineate a transient establishment phase in which the RCC integrates mechanical protection, coordinated turnover, and gravitropic control to promote robust root function during early growth.

## Summary statement

At germination, roots prioritise soil entry over long‐term foraging. We show that the transient root cap cuticle acts as a multifunctional organiser of this penetration‐optimised phase, integrating mechanical protection with organised root cap turnover and gravitropic control.

## 1 Introduction

Roots grow through soil to acquire water and nutrients, continually exposing the root tip to mechanical stress and potential tissue damage (Potocka and Szymanowska‐Pułka 2018). Therefore, sustained growth depends on mechanisms that preserve tissue integrity while retaining the ability to sense the surrounding environment and adjust growth direction accordingly (Berhin et al. 2019). These competing demands impose an inherent trade‐off, whereby a fully rigid protective barrier would compromise environmental sensing, whereas excessive exposure would increase vulnerability to damage. Plants resolve this trade‐off through the root cap, a multicellular structure positioned at the forefront of root–soil interactions (Dolan et al. 1993).

The root cap covers the root tip and forms the primary interface with the surrounding soil (Dolan et al. 1993). Moreover, the root cap protects meristematic tissues during growth through mechanically challenging substrates while remaining a major site for environmental perception and signal integration that steers directional growth, including gravity and moisture sensing and responses to mechanical cues at the root–soil interface (Juniper et al. 1966; Kobayashi et al. 2007; Zhu et al. 2025; Barlow 2002). Notably, the root cap maintains a dynamically renewed surface that provides both protection and sensory function, in which the outermost cell layers are shed and replaced in a regulated detachment–regeneration programme, enabling the tip to withstand mechanical damage while sustaining growth (Barlow 2002). This turnover cycle is coordinated by hormonal and redox signalling, including auxin and reactive oxygen species (ROS), to maintain root cap integrity during growth (Shin et al. 2025). Additionally, the secretion of pectic mucilage from the root cap provides local buffering against mechanical stress and facilitates root movement through the substrate (Iijima 2004). Together, this coordinated surface renewal and extracellular secretion provide protection while preserving sensory competence at the growing tip.

This strategy contrasts with that of aerial tissues, which do not require direct nutrient or water uptake from the external environment and can, therefore, rely on a stable epidermal cuticle (Yeats and Rose 2013). In shoots, the cuticle, composed of a cutin matrix with associated waxes, forms a hydrophobic diffusion barrier that limits non‐stomatal water loss and restricts pathogen entry, while also contributing to the mechanical robustness of the epidermis (Ingram and Nawrath 2017; Nawrath et al. 2013). Although changes in wax load and composition across tissues and environmental conditions can tune cuticular properties (Lee et al. 2025; Jeon et al. 2026), the primary function of the shoot cuticle remains protective.

Recent studies have revealed that a root cap cuticle (RCC) transiently forms on the outermost root cap cells (Berhin et al. 2019). At first glance, this cuticle‐like layer is counterintuitive since a hydrophobic barrier would be expected to compromise the balance between mechanical protection and environmental sensing at the root–soil interface. Therefore, this apparent paradox suggests that the RCC is unlikely to function solely as a mechanical barrier, but may instead serve additional roles specific to early root tip physiology. Indeed, the RCC has been reported to contribute to abiotic stress tolerance, such as protecting the root meristem under salt stress, as well as to facilitate lateral root emergence (Berhin et al. 2019; Uemura et al. 2024; Berhin et al. 2026). However, these reported roles do not readily explain the developmental specificity of the RCC. Unlike shoot cuticles, the RCC exists only briefly and is not regenerated after the first root cap detachment event (Berhin et al. 2019). Moreover, because salt stress can occur throughout root growth, the transient nature of the RCC suggests a function uniquely required during early root development.

Hence, this study aimed to address this possibility by examining the challenges faced by emerging seedlings and testing how the RCC contributes to early root penetration. Additionally, we assess whether this transient structure integrates mechanical protection with root cap turnover and directional control during the initial stages of root establishment. Consequently, we show that the RCC supports efficient initial penetration into dense substrates and is linked to orderly root cap detachment dynamics, including under non‐penetrating conditions. We further identified that changes in penetration competence along the seedling development track are coupled with gravitropic responsiveness, and that this coupling is disrupted in RCC‐deficient roots, whereas hydrotropic responses are largely preserved. These findings position the RCC as a transient, integrative element that coordinates early root cap performance during establishment.

## 2 Materials and Methods

### 2.1 Plant Material

All *Arabidopsis thaliana* lines examined in this study were derived from the Columbia‐0 (Col‐0) ecotype. Detailed descriptions of the Arabidopsis mutant and transgenic lines have been reported previously: *gpat4 gpat8* (Li et al. 2007), *bdg‐1* (Kurdyukov et al. 2006), *dcr‐2* (Panikashvili et al. 2009), *pin2* (Luschnig et al. 1998), *proDR5::GUS* (Ulmasov et al. 1997), *pin2 proDR5::GUS* (Ditengou et al. 2008).

### 2.2 Plant Growth Conditions

Seedlings were grown on half‐strength Murashige and Skoog (MS) medium supplemented with 1% (w/v) sucrose and solidified with agar. Seeds were surface sterilised by vapour‐phase sterilisation and stratified at 4°C for 2 days before sowing. Plants were grown under long‐day photoperiods, with light (70 μmol m⁻² s⁻¹) at 22°C for 16 h, followed by darkness at 18°C for 8 h. Agar concentrations of either 1.2% or 2.0% (w/v) were used, and seedlings were grown in either vertical or horizontal orientations as indicated.

### 2.3 Mechanical Impedance and Penetration Measurements

Two complementary agar‐based assays were performed to assess root penetration capacity. In assay 1, the upper portion of the agar medium was removed, and seeds were placed directly onto the exposed cut surface. Plates were maintained in a vertical orientation to allow root growth toward the agar surface and subsequent penetration into the agar. In assay 2, seeds were sown on intact agar surfaces and initially grown under vertical conditions. After primary root growth was established, plates were reoriented horizontally to promote root penetration into the agar matrix. Penetration efficiency was quantified as the proportion of germinated seedlings in which any part of the root penetrated into the agar medium, relative to the total number of germinated seedlings on the same plate, as previously described by Xu et al. (Xu et al. 2024).

### 2.4 Root Cap Cuticle Visualisation

The Fluorol Yellow 088 staining protocol was adapted from Berhin et al. (Berhin et al. 2019) with modified incubation conditions. Roots were incubated in 0.01% (w/v) Fluorol Yellow 088 in methanol for 4 days at room temperature in the dark. To reduce background fluorescence, samples were subsequently counterstained with 0.5% (w/v) aniline blue in water for 1 h, rinsed thoroughly, and imaged.

### 2.5 Root Cap Mucilage Visualisation and Pectinase Treatments

Root cap mucilage was visualised by staining with ruthenium red (0.005% [w/v]) for 1 min, followed by washes. Cell walls and nonviable cells were visualised by propidium iodide (PI) staining (10 μM, 2 min) followed by rinsing. For enzymatic perturbation, seedlings were grown on medium supplemented with 3.8 units of pectinase from *Aspergillus aculeatus*; controls received an equivalent volume of dimethyl sulfoxide (DMSO).

### 2.6 Root Cap Detachment Assay

Agar blocks containing intact seedlings were excised from the growth medium and transferred onto glass slides. For imaging, a small droplet of water was applied adjacent to the root apex before a cover slip was placed on the agar block. Root tips were imaged in bright‐field mode using an Axio Observer 5 microscope (Zeiss, Jena, Germany). Root cap detachment progression and detachment patterns were scored using criteria adapted from Shin et al. (Shin et al. 2025).

Detachment progression was classified into four stages: Initial, in which root cap cells were retained with visible amyloplasts; Stage 1, in which root cap cells remained attached but amyloplasts were no longer visible; Stage 2, in which shedding of the outermost root cap layer had begun, typically from lateral root cap cells; and Complete, in which the outermost root cap layer had been fully released, marking the end of one detachment event.

Detachment events were further categorised into three patterns: layered, in which a single outer root cap layer was shed as a continuous layer; unlayered, in which individual root cap cells detached independently; and multilayered, in which two or more root cap layers were released simultaneously.

### 2.7 Gravitropic Response Measurements

For the phenotypic analysis following gravistimulation, vertically grown seedling at 0‐, 3‐, and 7‐days after germination (DAG) were reoriented by 90°, and images were acquired 3 h later. To examine auxin distribution, *proDR5::GUS* reporter lines were subjected to gravistimulation and sampled immediately before and 20 min after reorientation. Seedlings were incubated in β‐glucuronidase (GUS) staining solution for 1 h and subsequently imaged in bright‐field mode using an Axio Observer 5 microscope (Zeiss, Jena, Germany).

### 2.8 Hydrotropic Response Measurements

For the hydrostimulation experiments, a split‐agar system was established to generate a moisture gradient. Square plates were first poured with half‐strength MS medium containing 1% (w/v) sucrose and 1% (w/v) agar. After solidification, the lower right portion of the agar was removed and replaced with half‐strength MS medium supplemented with 1% (w/v) sucrose, 1% (w/v) agar, and 400 mM D‐sorbitol. Seedlings at 2 or 6 DAG were placed on the split‐agar plates with their root tips positioned approximately 0.5 cm from the boundary between the two media. The plates were maintained in a vertical orientation for 24 h to allow directional root growth. After incubation, images were acquired, and root bending angles were quantified using Fiji (Schindelin et al. 2012).

### 2.9 Scanning Electron Microscopy (SEM)

For SEM analysis, plant tissues were prepared for ultrastructural integrity by critical‐point drying. Samples were dehydrated in a critical‐point dryer (EM CPD300, Leica, Vienna, Austria) and subsequently affixed to stainless steel specimen stubs with conductive carbon tape. To improve surface conductivity, mounted samples were sputter‐coated with platinum using a coating system (EM ACE200, Leica, Vienna, Austria). SEM observations were performed under high‐vacuum conditions using a JSM‐6390LV microscope (JEOL, Tokyo, Japan).

### 2.10 Microscopy and Imaging

Bright‐field imaging was performed using an Axio Observer 5 microscope (Zeiss, Jena, Germany). Confocal imaging was performed using an LSM 900/900 M laser‐scanning confocal microscope (Zeiss, Jena, Germany). PI was excited at 488 nm, and emission was collected at 550–720 nm. Fluorol Yellow 088 was excited at 488 nm and emission collected at 490–555 nm.

### 2.11 Quantification and Statistical Analysis

The statistical analyses employed are presented in the figure legends. Data were measured using ImageJ. Measurements for the phenotypic assays were statistically analysed in GraphPad Prism 10.5.0. Graphs were generated using GraphPad Prism 10.5.0.

## 3 Results

### 3.1 Root Cap Cuticle Facilitates Root Penetration Through Mechanical Protection

**Figure 1:**
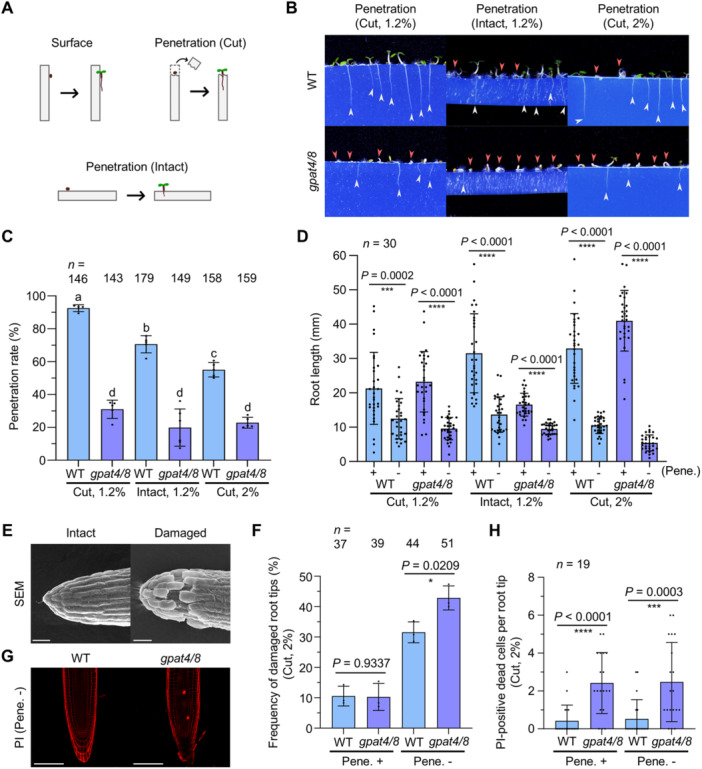
The root cap cuticle is required for penetration and protection from mechanical constraints. (A) Schematic diagram of the surface‐grown condition and two penetration assays. In the first assay, agar plates were positioned vertically, with the upper agar layer partially removed; seeds were placed on the exposed agar edge, allowing roots to grow downward and penetrate the medium. In the second assay, agar plates were positioned horizontally with intact agar, and roots were allowed to grow directly into and penetrate the solid medium. (B–D) Representative images showing penetration behaviour in 3‐DAG wild‐type (WT) and *gpat4 gpat8* (*gpat4/8*) mutant seedlings grown under the indicated conditions (B), together with quantification of penetration rate (C), and primary root length (D). White and red arrowheads indicate penetrating and non‐penetrating roots, respectively. Sample sizes: *n* > 142 roots for (C) and *n* = 30 roots for (D). (E) Representative scanning electron microscopy (SEM) images of the intact or mechanically damaged 3‐DAG root tips. (F) Quantification of frequency of damaged root tips based on SEM analysis in 3‐DAG WT and *gpat4/8* seedlings grown on 2% cut agar (*n* > 36 roots). Pene.+ indicates successful penetration, whereas pene.– indicates penetration failure. (G) Representative propidium iodide (PI)‐stained images of 3‐DAG WT and *gpat4/8* seedlings that failed to penetrate 2% cut agar. (H) Quantification of the total number of PI‐positive dead cells within the root tip in 3‐DAG WT and *gpat4/8* seedlings grown on 2% cut agar. Because damaged outer root cap surface cells are readily detached during the staining procedure, PI staining primarily reflects damage in the remaining root tip tissues rather than surface damage itself (*n* = 19 roots). All experiments were repeated at least three times; scale bars: 1 cm (B), 20 μm (E), and 100 μm (G). Statistical analyses used Brown–Forsythe and Welch analysis of variance (ANOVA) with Dunnett's T3 post hoc test (C), the unpaired t test with Welch's correction (D, F), and the Mann–Whitney test (H). Data are presented as the mean ± standard deviation (SD).

Efficient penetration of the surrounding substrate represents a primary challenge for emerging roots during early seedling establishment. Since penetration is governed by the mechanical properties of the growth interface, we first established agar‐based conditions that impose distinct constraints on root entry. Therefore, we compared cut agar, where the surface was incised to disrupt continuity and expose the inner porous structure, with intact agar, where the surface remained uninterrupted (Figure 1A). The wild‐type (WT) roots readily penetrated the 1.2% cut agar (~93%), whereas penetration was lower in 1.2% intact agar (~71%) and declined further in 2% cut agar (~55%) (Figure 1B,C), thereby supporting the view that surface continuity and overall stiffness impose a major constraint on root entry.

Roots that failed to penetrate showed pronounced growth retardation (Figure 1D). SEM revealed increased surface damage in these roots (Figure 1E,F), indicating that penetration failure is associated with enhanced mechanical damage at the root tip. In contrast, roots that successfully penetrated exhibited comparable post‐penetration growth across conditions (Figure 1D), indicating that, once penetration occurs, subsequent growth proceeds similarly across substrate conditions.

Having established assay conditions that impose distinct mechanical constraints on root entry, we next tested the contribution of the RCC. We analysed the glycerol‐3‐phosphate acyltransferase mutant *gpat4 gpat8*, which exhibits impaired RCC formation (Berhin et al. 2019). To exclude any potential effect of germination differences on analysis, penetration efficiency was assessed only among germinated seedlings; *gpat4 gpat8* showed a slightly lower germination rate than WT (Figure S1A). Under these conditions, *gpat4 gpat8* exhibited lower penetration efficiency than WT across all tested cut agar conditions, with penetration rates of approximately 31% versus 93% on 1.2% cut agar and 23% versus 55% on 2% cut agar (Figures 1C and S1B). Although surface‐grown *gpat4 gpat8* seedlings were only slightly shorter than WT on 1.2% agar (Figure S1C), root growth was much more strongly reduced when penetration failed. On 2% cut agar, non‐penetrating roots measured approximately 5.4 mm in *gpat4 gpat8*, compared with 10.5 mm in WT (Figure 1D). These observations raised the possibility that the RCC contributes not only to penetration efficiency, but also to maintaining root tip integrity under mechanical stress during penetration.

We next examined whether the RCC contributes to maintaining root tip integrity during penetration. SEM analysis showed that root tips grown on the agar surface were largely intact, whereas damage became evident during penetration and was most severe in roots that failed to penetrate (Figures 1F and S2). The frequency of damaged root tips was higher in *gpat4 gpat8* than in WT, particularly among non‐penetrating roots, while penetrated roots showed comparable levels of damage between the two genotypes (Figures 1F and S2). To assess damage throughout the root tip region, we next performed PI staining (Figure 1G). Because damaged outer root cap surface cells are readily detached during staining, PI staining primarily reflects damage in the remaining root tip tissues rather than surface damage itself. Quantification of PI‐positive dead cells under cut 2% agar conditions showed a clear increase in *gpat4 gpat8* relative to WT (Figure 1H). These results indicate that the RCC is important not only for maintaining outer surface integrity during penetration, but also for protecting inner root tip tissues.

### 3.2 Enhanced Mucilage Buffers RCC Loss During Root Penetration

**Figure 2:**
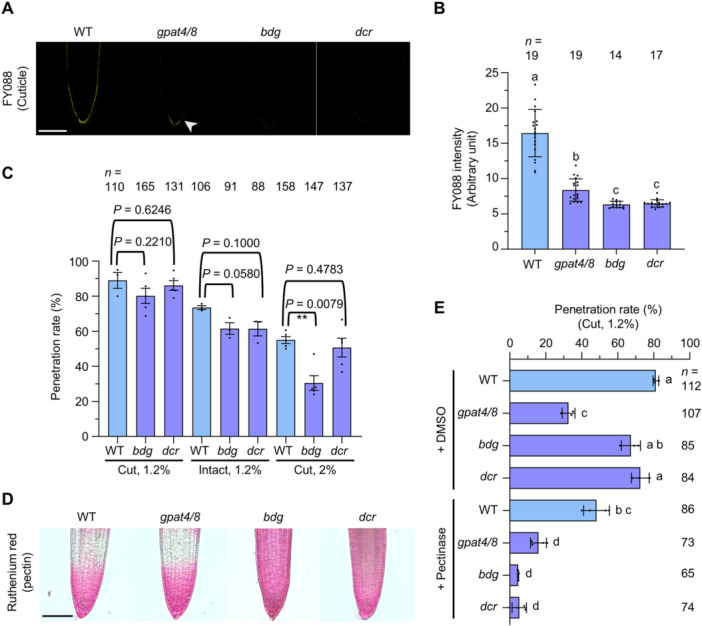
Compensatory mucilage secretion supports penetration in root cap cuticle‐deficient *bdg* and *dcr* mutants. (A, B) Representative images (A) and quantification (B) of cuticle staining using Fluorol Yellow 088 (FY088) in 3‐DAG seedlings grown under surface growth conditions (*n* > 13 roots). The white arrowhead indicates root tip‐specific cuticle deposition observed in the *gpat4/8* mutants. (C) Quantification of penetration rates in 3‐DAG wild‐type (WT), *bdg*, and *dcr* seedlings grown under different conditions (*n* > 87 roots). (D) Representative images of ruthenium red staining for pectin in 3‐DAG roots (*n* = 30 roots). (E) Quantification of changes in penetration rates in response to pectinase treatment in 3‐DAG seedlings (*n* > 64 roots). All experiments were repeated at least three times: scale bars, 100 μm (A, D). Statistical analyses used Brown–Forsythe and Welch ANOVA with Dunnett's T3 post hoc test (B, E), the Mann–Whitney test for the *dcr* intact 1.2% group and the *bdg* cut 2% group in (C), and unpaired t tests with Welch's correction for the remaining groups in (C). Data are presented as the mean ± SD. [Color figure can be viewed at wileyonlinelibrary.com]

To further examine the contribution of the RCC to root penetration, we extended our analysis to additional cuticle‐deficient mutants, *bodyguard* (*bdg*) and *defective in cuticular ridges* (*dcr*). Consistent with previous reports (Berhin et al. 2019), Fluorol Yellow 088 (FY 088) staining revealed more severe defects in RCC formation in the *bdg* and *dcr* mutants than in the *gpat4 gpat8* mutant, in which a partial columella cuticle remained detectable (Figure 2A,B).

However, penetration performance did not scale with RCC abundance. In both the cut and intact 1.2% agar conditions, the *bdg* and *dcr* roots penetrated at efficiencies comparable to WT; a significant reduction was observed in the *bdg* roots only for the 2% cut agar (Figure 2C). Notably, this reduction was less severe than that observed for the *gpat4 gpat8* mutant (Figure 1C). These results indicate that penetration efficiency is not determined solely by residual cuticle levels and suggest that additional factors can buffer RCC loss.

Therefore, we investigated whether compensatory mechanisms are active in mutants deficient in RCC. Previous immunolabeling with a xylogalacturonan‐specific antibody revealed enhanced mucilage accumulation in the *bdg* and *dcr* mutant roots (Berhin et al. 2019). In agreement, ruthenium red staining, which labels acidic polysaccharides enriched in pectin‐based mucilage, produced strong signals in the *bdg* and *dcr* mutant roots but not in WT or *gpat4 gpat8* (Figure 2D). Next, to test whether elevated mucilage contributes functionally to penetration in RCC‐null backgrounds, we quantified penetration efficiency after pectinase treatment under conditions that did not induce detectable cell death (Figure S3A,B). Enzymatic removal of pectin significantly reduced penetration in *bdg* and *dcr*, with the strongest reduction in the most RCC‐deficient backgrounds (Figure 2E). Together, these results indicate that enhanced mucilage secretion in RCC‐null mutants functionally compensates for cuticle loss, likely improving lubrication and local buffering at the root–substrate interface to support efficient root penetration.

However, efficient penetration was not always accompanied by equivalent protection of the root tip. Although *bdg* penetrated more efficiently than *gpat4 gpat8*, it showed frequencies of damaged root tips and PI‐positive cells similar to those of *gpat4 gpat8* (Figures S2 and S3C,D). By contrast, *dcr* exhibited not only high penetration efficiency but also WT‐like frequencies of damaged root tips and PI‐positive cells (Figures S2 and S3C,D). These results indicate that although compensatory mucilage promotes penetration in both *bdg* and *dcr*, its consequences for root tip protection differ between genotypes.

### 3.3 RCC Coordinates Root Cap Detachment Dynamics

**Figure 3:**
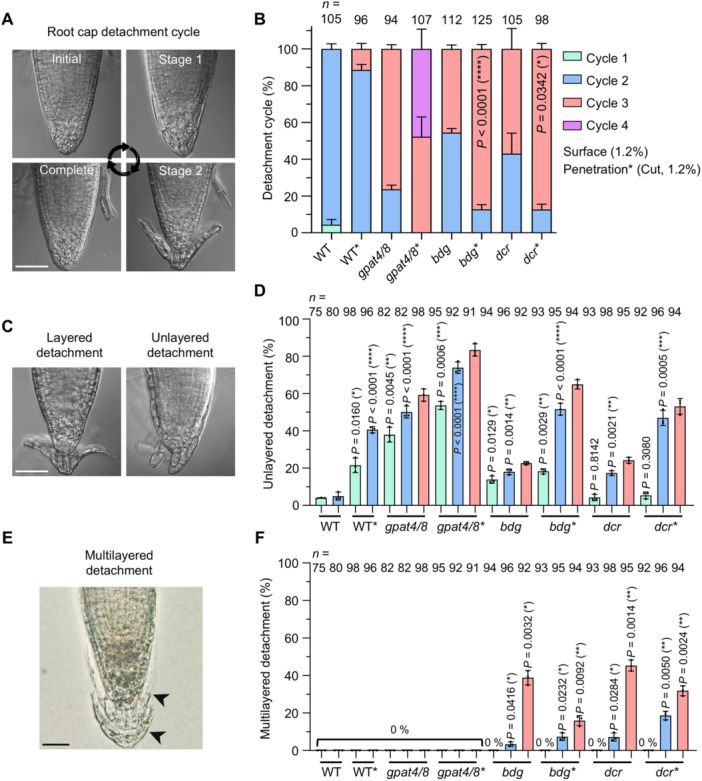
Effects of penetration and the root cap cuticle on the root cap detachment. (A) Representative images of the initial state, stage 1, stage 2, and complete detachment during the root cap detachment cycle. (B) Quantification of the root cap detachment cycle in 5‐ and 6‐DAG seedlings of WT, *gpat4 gpat8* (*gpat4/8*), *bdg*, and *dcr* grown on 1.2% agar under either surface or cut‐penetration conditions. For the cut‐penetration condition, only seedlings that successfully penetrated the agar surface were analysed (*n* > 93 roots). (C, D) Representative images of layered and unlayered detachment (C) and quantification of these detachment patterns in 3‐ to 6‐DAG seedlings of different genotypes grown on 1.2% agar under surface or cut‐penetration conditions (D) (*n* > 74 roots). (E, F) Representative images of multilayered and unlayered detachment (E) and quantification of these detachment patterns in 3‐ to 6‐DAG seedlings of different genotypes grown on 1.2% agar under surface or cut‐penetration conditions (F) (*n* > 74 roots). Quantification of the root cap detachment cycle was performed using independent experimental sets, whereas unlayered, layered, and multilayered detachment were quantified within the same experimental groups. All experiments were repeated at least three times: scale bars, 50 μm (A, C, E). Statistical analyses were performed using unpaired t tests with Welch's correction (B, D, F). In (B), surface‐grown and penetration‐grown conditions were compared. In (D), each mutant was compared with WT seedlings at the same growth cycle. In (F), each genotype was compared with the corresponding cycle 1 condition. Data are presented as the mean + SD (B) and mean ± SD (D, E). [Color figure can be viewed at wileyonlinelibrary.com]

Penetration is not achieved by a static, shielded tip alone. The root cap surface must be renewed in a coordinated manner, removing compromised cells while preserving an organised, functional interface for continued growth (Figure 3A) (Shin et al. 2025). Given that the RCC forms on the outermost root cap layers, we evaluated whether the RCC contributes to both mechanical protection and the timing and collective organisation of root cap detachment.

We first quantified how penetration affects detachment dynamics in WT roots. Under surface growth conditions, most WT roots had progressed to detachment cycle 2—completion of the first detachment event—by 5 to 6 days after germination (DAG) (Figures 3B and S4). WT roots progressed further under conditions that impose penetration, with ~11% reaching cycle 3 (Figures 3B and S4), indicating that penetration can accelerate progression through the detachment cycle.

Next, we analysed the RCC‐defective mutants under the same experimental conditions. The RCC‐defective mutants detached more rapidly than WT even on the agar surface, with an increased frequency by cycle 3, indicating that RCC loss accelerates detachment independently of penetration (Figure 3B and S4). Moreover, acceleration was further enhanced under penetration conditions: the *gpat4 gpat8* roots frequently reached cycle 4, whereas the *bdg* and *dcr* roots showed elevated frequencies at cycle 3 (Figure 3B). Thus, penetration and RCC loss act additively to advance the detachment cycle, with the magnitude of acceleration reflecting the functional state of the root cap surface.

In addition to altering the timing of root cap turnover, the mechanical challenge imposed by penetration also affected the structural organisation of detachment. In WT seedlings grown on the surface, the outermost root cap cells typically detached as a cohesive sheet (Figure 3C,D). Meanwhile, under penetration conditions, this organised pattern was disrupted from the first cycle onward, with the outermost cells separating individually or as fragmented units rather than forming an intact layer; disorganisation became more pronounced during the second cycle, when cuticle regeneration no longer occurs (Figure 3D).

Disorganised detachment was evident in RCC‐defective mutants even under surface conditions. This phenotype was particularly pronounced in the *gpat4 gpat8* mutant, which lacks mucilage‐based compensation, with up to ~38% of roots exhibiting disorganised detachment (Figure 3D). Notably, the frequency of disorganised detachment increased over successive cycles, suggesting that defects arising during the initial detachment event persist and influence later stages when cuticle regeneration no longer occurs. In contrast, *bdg* and *dcr* frequently exhibited multilayered detachment under both surface and penetration conditions, with severity increasing over successive cycles (Figure 3E,F), potentially reflecting excessive or misregulated mucilage accumulation.

Together, these results reveal that the RCC regulates both the timing and the collective organisation of detachment of the outermost root cap cells. Notably, the loss of the RCC accelerates and disorganises detachment even under surface conditions, indicating that RCC status shapes the turnover programme ahead of physical entry into the substrate, with defects initiated at the first detachment event persisting across subsequent cycles.

### 3.4 RCC Supports Gravitropic Responsiveness During Early Root Penetration

**Figure 4:**
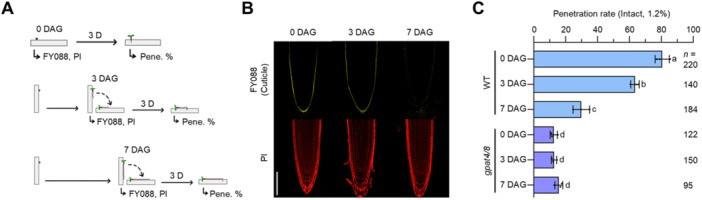
An age‐dependent decline in penetration rate is observed in wild‐type but not in the *gpat4 gpat8* mutant. (A) Schematic diagram showing the timing of Fluorol Yellow 088 (FY088) and PI staining, and the assessment of penetration rates after reorienting the growth medium from vertical to horizontal at 0, 3, and 7 DAG. (B) Representative FY088 and PI staining images of seedlings grown under surface growth conditions (*n* > 20 roots). Scale bar, 100 μm. (C) Quantification of penetration rates in WT and *gpat4 gpat8* (*gpat4/8*) seedlings (*n* > 94 roots). Statistical analyses were performed using one‐way ANOVA followed by Tukey's post hoc test. [Color figure can be viewed at wileyonlinelibrary.com]

While the RCC facilitates penetration into mechanically resistant media, cuticle formation ceases once the first root cap detaches (Figure 4A). Therefore, to examine how this developmental change affects penetration capacity, seedlings were initially grown on the agar surface for varying durations. Subsequently, the plates were tilted by 90° and the roots were allowed to grow into the medium for 3 days (Figure 4B). Under these conditions, penetration efficiency was markedly reduced in 7‐day‐old seedlings, in which the RCC is no longer present (Figure 4C), consistent with reduced penetration competence following cuticle loss. However, penetration efficiency was already lower at 3 DAG than at 0 DAG, despite the continued presence of the cuticle, indicating that additional age‐dependent factors contribute to the progressive decline in penetration capacity. This age‐dependent decline was not observed in the *gpat4 gpat8* mutant, in which penetration efficiency remained uniformly low across developmental stages (Figure 4C), consistent with an early and persistent defect associated with impaired RCC formation.

**Figure 5:**
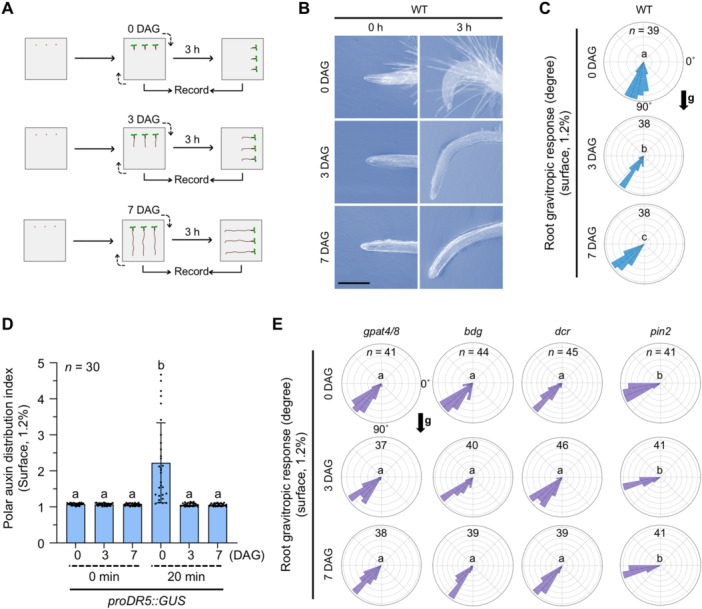
Root cap cuticle affects gravitropic response. (A) Schematic diagram of the reorientation experiment performed at 0, 3, and 7 DAG. (B, C) Representative images of root gravitropic responses before and 3 h after reorientation (B) and quantification of the gravitropic response (C) under the indicated conditions and time points (*n* > 37 roots). Scale bar, 500 μm. (D) Quantification of relative auxin distribution in WT seedlings before reorientation and 20 min after a 90° gravistimulus. The degree of asymmetric *proDR5::GUS* signal distribution between opposing sides of the root was measured to assess auxin redistribution after reorientation (*n* = 30 roots). (E) Quantification of gravitropic responses in the root cap cuticle mutants *gpat4 gpat8* (*gpat4/8*), *bdg*, and *dcr*, and in the gravitropic response mutant *pin2*, under the indicated conditions and time points (*n* > 36 roots). All experiments were repeated at least three times. Statistical analyses used Brown–Forsythe and Welch ANOVA with Dunnett's T3 post hoc test (C) and Kruskal–Wallis test with Holm correction followed by Dunn's post hoc test (D, E). Data are presented as circular histograms in (C, E) and as mean ± SD in (D). [Color figure can be viewed at wileyonlinelibrary.com]

To identify age‐dependent factors beyond the cuticle, we focused on the gravitropic response. Since penetration into the medium requires rapid reorientation of the root tip in response to gravistimulation, we considered that a developmental weakening of gravitropic responsiveness might underlie the progressive loss of penetration capacity. This proved to be the case: gravitropic curvature declined sharply with age, decreasing from ~80° in freshly germinated roots to ~56° at 3 DAG and < 41° at 7 DAG (Figure 5A–C). This developmental decline in gravitropism was further supported by altered auxin distribution patterns in older roots (Figures 5D and S5). In contrast, the RCC mutants showed no age‐dependent changes in gravitropic response (Figure 5E). Although the gravitropic response in RCC mutants was less severely impaired than in the *pin2* mutant (Figure 5E), these mutants consistently displayed reduced gravitropism across developmental stages, regardless of mucilage presence, indicating that the RCC contributes to optimal gravitropic responsiveness.

**Figure 6:**
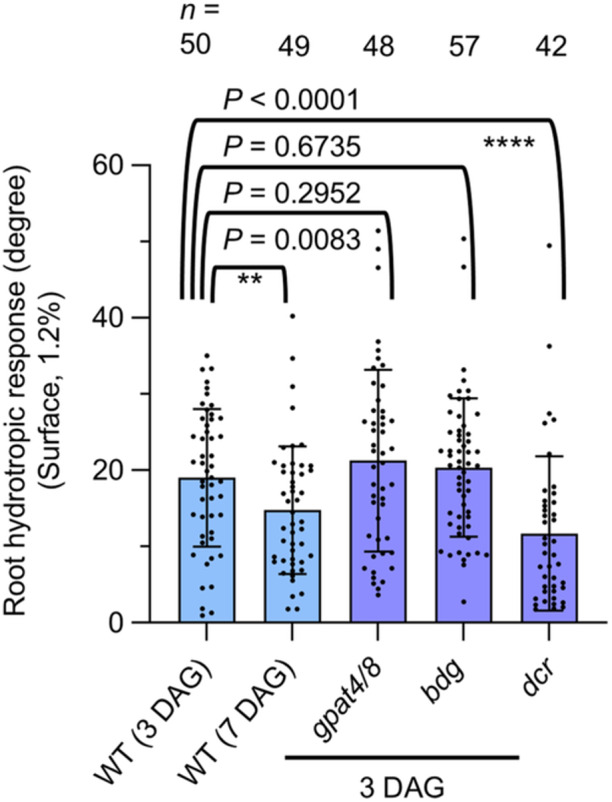
The root cap cuticle is not required for the hydrotropic response. Quantification of root hydrotropic curvature in WT, *gpat4 gpat8* (*gpat4/8*), *bdg*, and *dcr* seedlings after 1 day of treatment with 400 mM D‐sorbitol at the indicated day. The experiment was repeated three times. Statistical analyses were performed using unpaired *t* tests with Welch's correction for *gpat4/8* and the Mann–Whitney test for the other genotypes. Data are presented as mean ± SD. [Color figure can be viewed at wileyonlinelibrary.com]

Alongside the decline in gravitropism, hydrotropic responses also decreased with age in WT roots (Figure 6). However, no substantial differences were observed among WT, *gpat4 gpat8*, and *bdg*, whereas the *dcr* mutant showed a tendency toward a reduced response. These observations indicate that the RCC function is less tightly linked to hydrotropism than to gravitropism. Together, these findings suggest that the early root tip represents a developmental state optimised for penetration, supported by a robust gravitropic response and a protective cuticle.

## 4 Discussion

**Figure 7:**
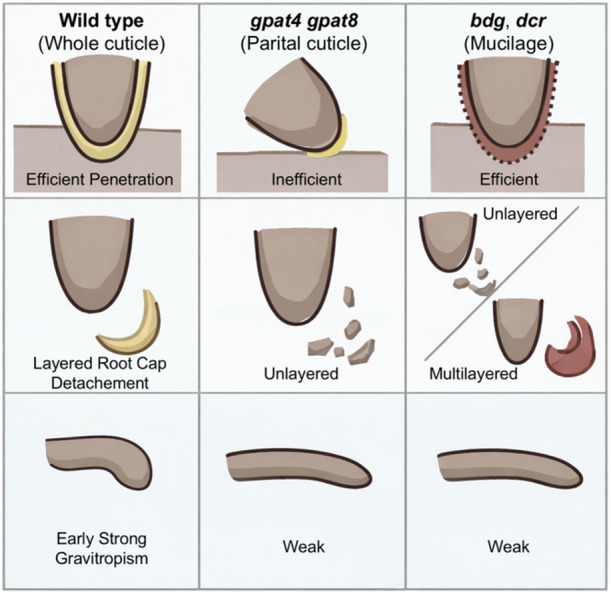
Summary model of the role of the root cap cuticle in early root penetration. In WT roots, an intact root cap cuticle (RCC) provides mechanical protection at the root tip, supports organised, layered root cap detachment, and enables efficient penetration accompanied by strong early gravitropic responses. In *gpat4 gpat8* mutants, a partial loss of the RCC compromises mechanical protection, leads to unlayered root cap detachment, and results in inefficient penetration and weakened gravitropism. In contrast, *bdg* and *dcr* mutants, which are characterised by mucilage accumulation rather than defects in cuticle integrity, retain penetration efficiency despite altered unlayered or multilayered root cap detachment patterns, but exhibit reduced gravitropic responses similar to those observed in *gpat4 gpat8*. Together, this model illustrates that the RCC defines a transient, penetration‐optimised state of the root tip through the integration of mechanical robustness, controlled root cap turnover, and gravitropic competence. [Color figure can be viewed at wileyonlinelibrary.com]

Roots sustain growth in soil by continuously sensing the surrounding environment, coping with mechanical pressure, and adjusting development to optimise water and nutrient acquisition. However, for germination, these longer‐term functions depend on an immediate, stage‐specific prerequisite: the emerging root must rapidly achieve reliable soil entry. Nonetheless, the mechanism through which the root tip is configured to meet this early priority has previously remained unclear. Notably, this study demonstrates that the RCC defines an early, penetration‐optimised root tip state (Figure 7). In addition to providing mechanical protection, the RCC coordinates the timing and collective organisation of root cap detachment while sustaining robust gravitropic responsiveness, thereby promoting effective soil entry at the onset of development.

Our penetration assays suggest that the first breach constitutes a mechanically demanding bottleneck for the root tip. Thus, as the mechanical resistance increased, the surface damage rose sharply and was most evident in roots that failed to penetrate, whereas growth after successful entry was comparatively insensitive to these differences in resistance. Together, these patterns indicate that the dominant mechanical challenge is concentrated at the moment of entry. These findings are consistent with a stage‐specific division of labour in which plants deploy distinct surface systems before and after soil entry. During the initial breach, a stiffer and more wear‐resistant surface layer, such as the RCC, may be required to resist localised compression and abrasion that cannot be fully buffered by mucilage alone. After entry, pectin‐rich mucilage may confer greater benefits by dissipating stress through hydration and viscoelastic damping, and by reducing friction at the root–substrate interface (Iijima 2004). In line with this view, enhanced pectin accumulation in the *bdg* and *dcr* mutants improved penetration efficiency yet did not fully prevent cellular damage under penetration‐imposing conditions. Together, our results provide a functional rationale for why the root tip employs distinct surface barriers across establishment: the RCC protects the tip during the mechanically extreme first breach, whereas pectin‐rich mucilage is better suited for buffering and lubrication during subsequent growth through the substrate. These interpretations should nevertheless be considered with caution. Root penetration efficiency may also be influenced by root growth itself, and the relative contributions of growth capacity and surface properties remain to be resolved. Moreover, because the pectinase treatment applied here is expected to affect not only RCC‐associated mucilage but also cell wall pectin more broadly, the present results do not isolate the specific contribution of mucilage alone. Direct genetic analysis will therefore be required to test the compensatory role of mucilage in RCC‐null backgrounds. In particular, combining cuticle‐deficient mutants with mutants impaired in mucilage production, such as *brn2* (Liu et al. 2024), may provide a useful framework for dissecting the respective contributions of the RCC and mucilage.

In addition to supporting penetration, our results show that the RCC influences root cap detachment dynamics. Indeed, RCC loss accelerated detachment progression even in surface‐grown roots before mechanical stress imposed by penetration, indicating that the altered detachment is not simply a passive consequence of increased external pressure. Importantly, this earlier progression persisted beyond the first detachment event, when the newly formed root cap no longer carries a cuticle, suggesting that early RCC status imprints on the detachment programme and helps establish the timing of turnover early in development.

The accompanying changes in detachment organisation further support a role beyond stress buffering. In the RCC mutants, the outermost cells are often separated in a disorganised manner rather than forming a cohesive sheet, suggesting that a continuous cuticular layer influences the distribution of forces across the detaching surface. Mechanical stress applied to a mechanically coupled sheet is expected to dissipate more evenly than stress borne by individual cells detaching independently. Thus, mechanical protection, force distribution, and collective detachment behaviour are closely linked, providing a mechanistic basis for the deployment of a cuticle‐based surface at the earliest stage of root cap development.

Notably, the process through which the RCC status is perceived and translated into detachment timing remains unclear. However, one possibility is that altered mechanics or adhesion at the outermost root cap layer modulate upstream signalling that governs the turnover cycle. In this context, recent work has identified ROS as oscillatory regulators acting upstream of auxin to control root cap detachment dynamics (Shin et al. 2025; Dubreuil et al. 2018). Hence, exploring potential interactions between RCC‐dependent surface properties, ROS dynamics, and auxin signalling may provide valuable insight into how detachment timing is established and maintained during early root development.

Another defining feature of this early, penetration‐optimised root tip state is unusually strong gravitropic responsiveness immediately after germination. We observed that gravitropic curvature declines with seedling age, consistent with a transition from an early, strongly gravitropic mode to a more integrated growth strategy that balances gravity with other heterogeneous environmental cues. The initial high directional bias is likely advantageous for rapid, downward‐directed growth, minimising deviations and promoting efficient soil entry during the narrow window when penetration is critical for seedling survival. Notably, the RCC was essential for this early gravitropic competence: RCC‐deficient mutants exhibited persistently reduced gravitropic responses across developmental stages. Given that both gravitropic regulation and root cap detachment rely heavily on auxin signalling (Dubreuil et al. 2018; Su et al. 2017), RCC‐dependent modulation of the root cap surface properties may act through a shared upstream framework that coordinates directional growth and turnover dynamics. Therefore, defining how surface properties contribute to auxin‐mediated regulation will be important for understanding how this early, penetration‐optimised state is established.

In contrast to the pronounced effects on gravitropic responsiveness, hydrotropic responses were largely preserved in the RCC‐defective mutants, with the *gpat4 gpat8* and *bdg* mutants showing no substantial differences from WT roots. This distinction is consistent with current models of hydrotropism, which emphasise the involvement of abscisic acid (ABA) signalling and growth regulation in the elongation and transition zones rather than auxin‐driven polarity changes in the root cap (Shkolnik et al. 2016; Dietrich et al. 2017). A modest reduction was observed in the *dcr* mutants; however, this effect did not correlate with RCC deficiency or mucilage overproduction, suggesting a *dcr*‐specific contribution independent of RCC function. Overall, these results support a selective link between the RCC and gravity‐driven directional growth during early root development, while hydrotropic responses remain largely intact.

From an evolutionary perspective, a transient, penetration‐optimised root tip may represent an adaptive solution to the high‐risk period immediately following germination. Our findings redefine the RCC as a multifunctional, transient surface structure that integrates mechanical protection, tissue organisation, and environmental responsiveness during this early window. By prioritising mechanical robustness and gravity‐driven directional growth before transitioning toward broader environmental responsiveness, seedlings may maximise the likelihood of successful establishment in heterogeneous soil environments (Zhang et al. 2019; Berhin et al. 2019). More broadly, our study highlights how transient extracellular structures can exert disproportionate control over developmental robustness and early environmental adaptation, offering a framework for understanding how plants establish growth competence at the onset of soil exploration.

## Conflicts of Interest

The authors declare no conflicts of interest.

## Supporting information

Supporting File

## Data Availability

The data that support the findings of this study are available from the corresponding author upon reasonable request.
