# Supplementary material for: Root Cap Cuticles Confer a Transient, Penetration‐Optimised Phase During Early Seedling Establishment
Source: Plant Cell Environ. 2026 May 22;49(9):6384–95. doi: 10.1111/pce.70622 (PMC13436564; doi:10.1111/pce.70622)
Supplement: Supplementary file 1 [file PCE-49-6384-s001.pdf]

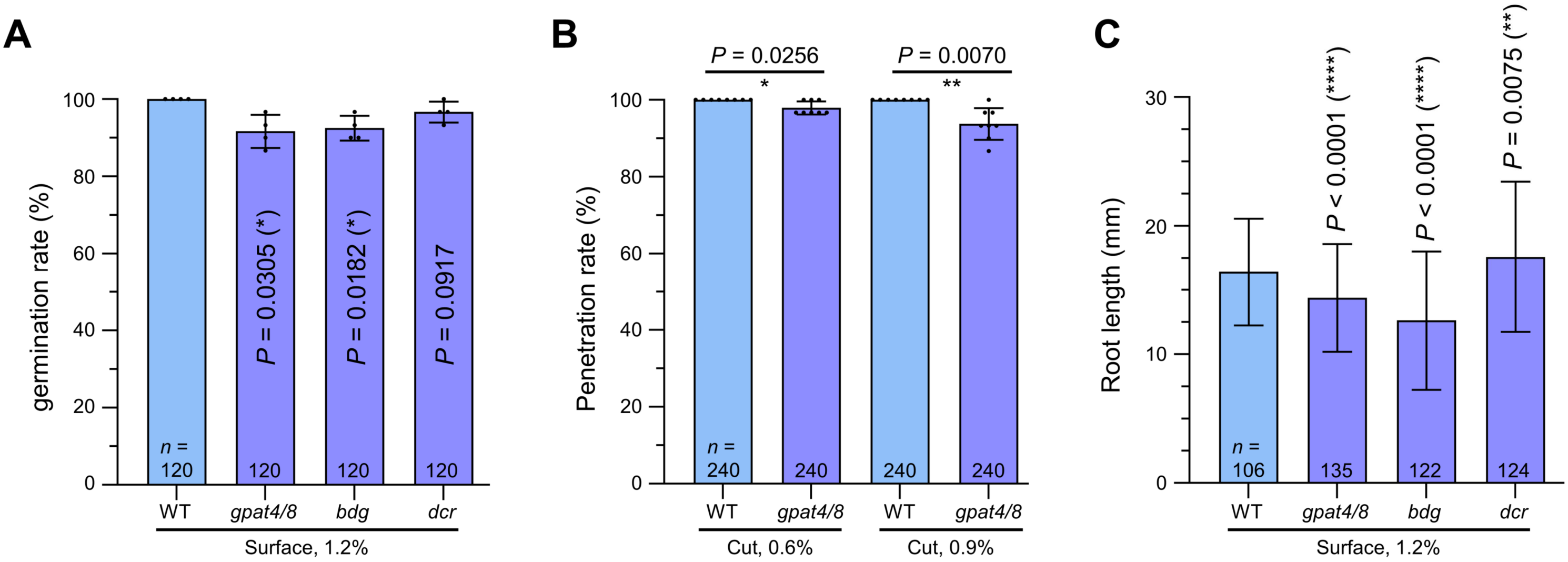


**Fig. S1. Germination, root growth, and penetration phenotypes of root cap cuticle mutants.**

(A) Germination rates of wild-type (WT) and the root cap cuticle mutants *gpat4 gpat8* (*gpat4/8*), *bdg*, and *dcr*, measured at 2 days after sowing, prior to penetration rate analysis ($n$ = 120 roots).

(B) Penetration rates of 3-DAG WT and *gpat4/8* seedlings grown under cut medium conditions containing 0.6% or 0.9% agar ($n$ = 240 roots).

(C) Measurement of primary root length of 3 DAG seedlings of WT, *gpat4/8*, *bdg*, and *dcr* ($n > 100$ roots).

All experiments were repeated at least three times. Statistical analysis was performed using unpaired t-tests with Welch's correction (A) and the Mann–Whitney test (B, C). For (A) and (C), each genotype was compared with WT. Data are presented as mean ± SD.

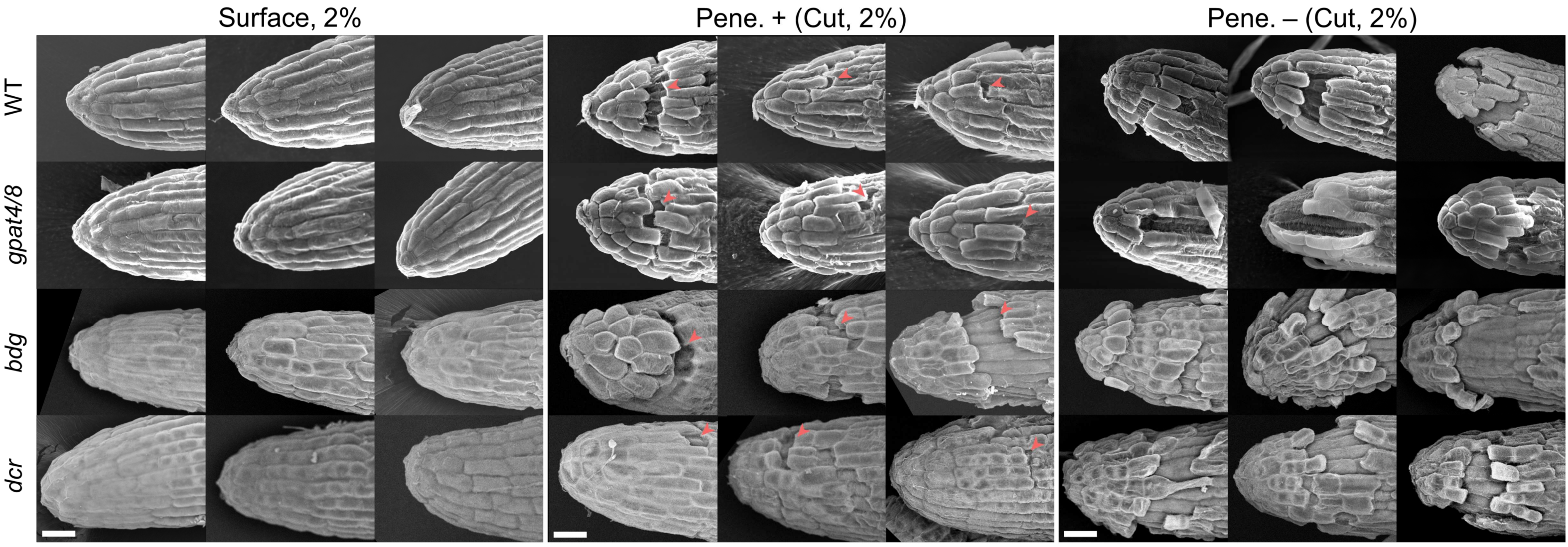


**Fig. S2. Root tip damage associated with penetration outcome in WT and root cap cuticle mutants.** Representative scanning electron microscopy images of root tips from 3-DAG seedlings grown on 2% agar either on the surface or under cut conditions. Three independent root tips are shown for each genotype and condition. Pene.+ indicates successful penetration, whereas Pene.– indicates failure to penetrate. Arrowheads indicate damaged regions. More than 20 roots were examined across three independent experiments. Scale bars, 20 μm.

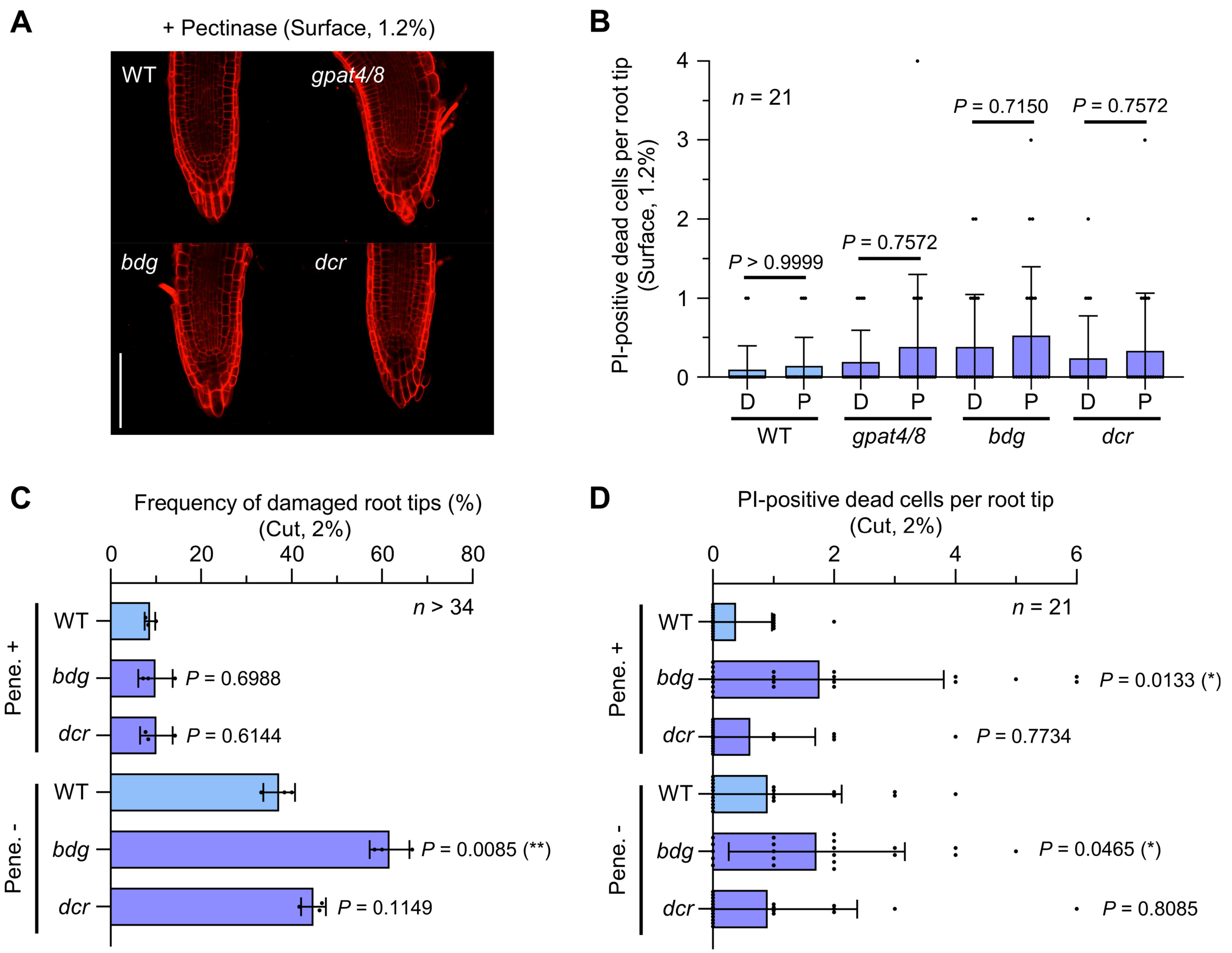


**Fig. S3. Root tip damage and cell death patterns in root cap cuticle mutants under mechanical constraint and pectinase treatment.**

(A, B) Representative propidium iodide (PI)-stained images (A) and quantification of PI-positive dead cells (B) in 3-DAG WT, *gpat4/8*, *bdg*, and *dcr* seedlings grown on the surface of 1.2% agar ($n = 21$ roots). D indicates DMSO treatment, and P indicates pectinase treatment.

(C) Quantification of the frequency of damaged root tips based on scanning electron microscopy (SEM) analysis in 3-DAG WT, *bdg*, and *dcr* seedlings grown on 2% cut agar ($n$ = 35–42 roots). Pene.+ indicates successful penetration, whereas Pene.– indicates penetration failure.

(D) Quantification of PI-positive dead cells within the root tip in 3-DAG WT, *gpat4/8, bdg*, and *dcr* seedlings grown on 2% cut agar ($n = 21$ roots). Damaged outer root cap surface cells are readily detached during staining and are therefore underrepresented in this analysis.

All experiments were repeated at least three times; scale bars: 100 µm (A). Statistical analyses were performed using the paired t-test (C) and the Mann–Whitney test (B, D). For C and D, each genotype was compared with WT.

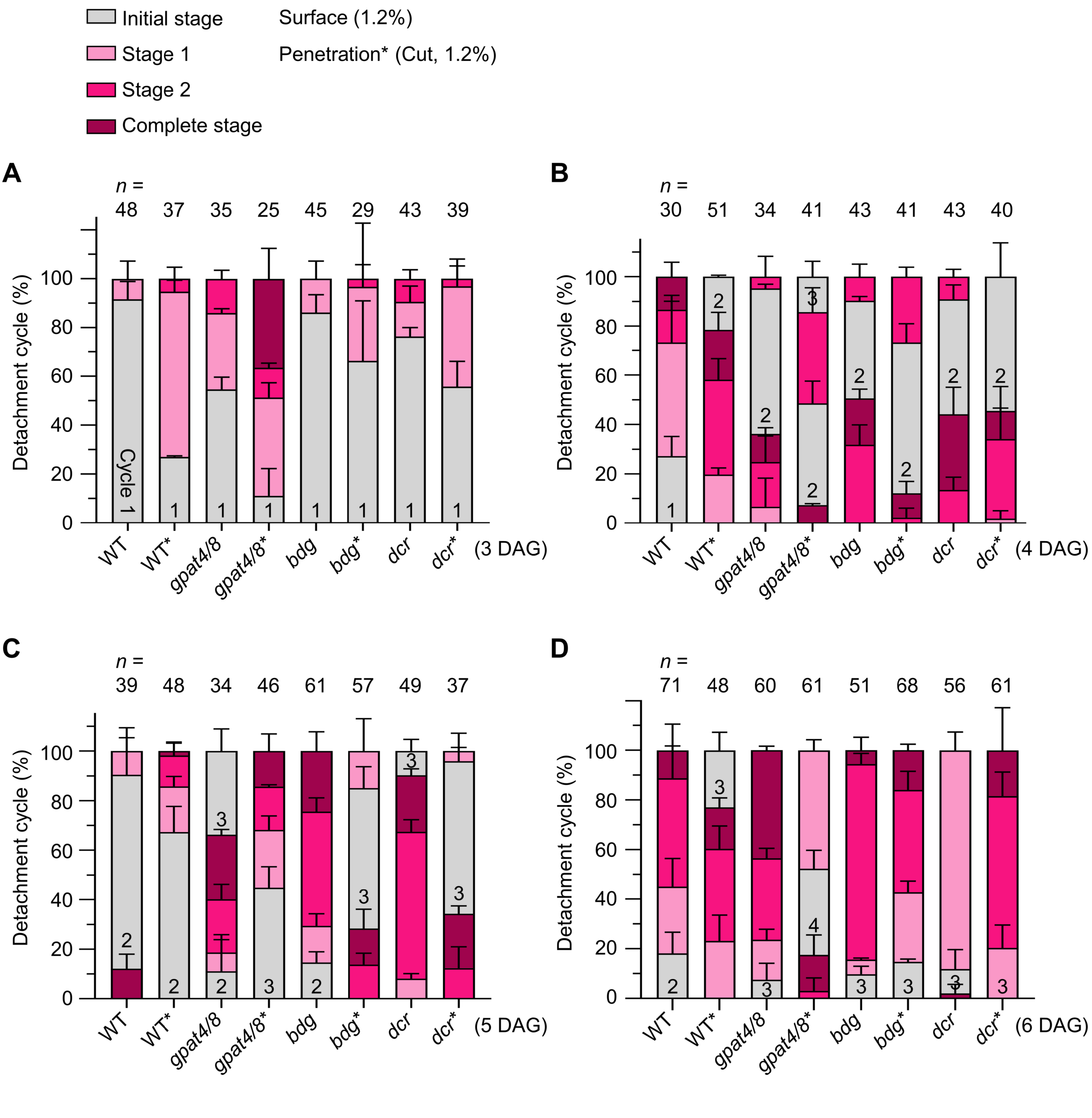


**Fig. S4. Time-resolved root cap detachment cycles in wild-type and root cap cuticle-deficient mutants.** The root cap detachment cycle was quantified in wild-type (WT), *gpat4 gpat8* (*gpat4/8*), *bdg*, and *dcr* seedlings at 3, 4, 5, and 6 DAG, grown under surface-growth or cut-penetration conditions on 1.2% agar ($n > 24$ roots). For the cut-penetration group, only seedlings that successfully penetrated the agar surface were analyzed. Individual detachment cycles are indicated by the numbers within the graphs. For each numbered cycle, the data shown above corresponds to the stated cycle, whereas the data shown below represents the preceding cycle. Data are presented as the mean + standard deviation (SD).

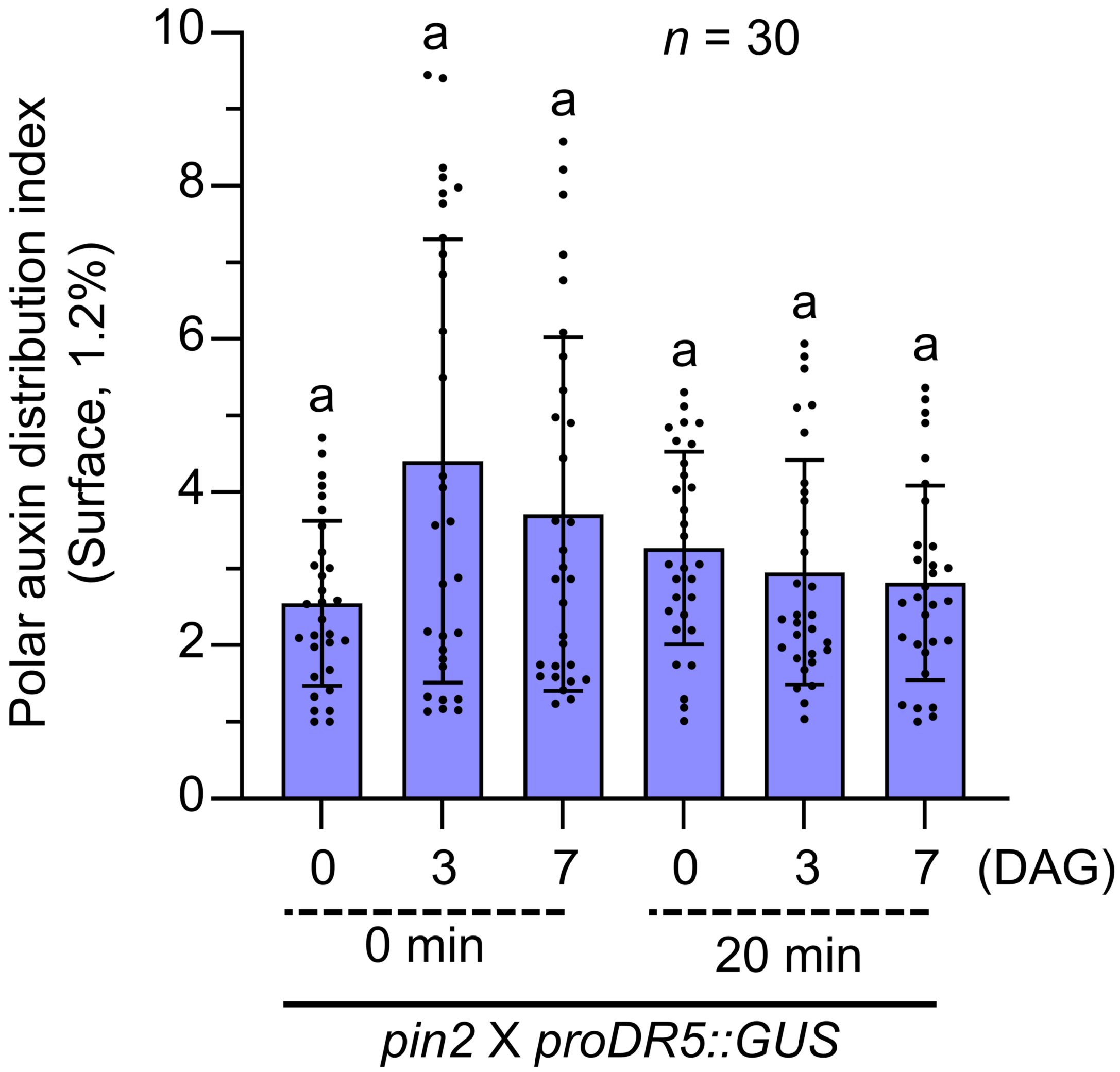


**Fig. S5. Age- and reorientation-dependent changes in *DR5::GUS* activity in the *pin2* mutant line.** Relative auxin distribution was quantified in *pin2* seedlings at 0, 3, and 7 DAG, both before reorientation and 20 minutes after a 90° gravistimulus ($n$ = 30 roots). The degree of asymmetric *proDR5::GUS* signal distribution between the opposing sides of the root was measured to assess auxin redistribution after reorientation. Statistical analysis was performed using the Kruskal–Wallis with Dunn's multiple comparisons test. Data are presented as the mean ± SD.
